# Investigating an Airborne Tularemia Outbreak, Germany

**DOI:** 10.3201/eid1602.081727

**Published:** 2010-02

**Authors:** Anja M. Hauri, Iris Hofstetter, Erik Seibold, Philip Kaysser, Juergen Eckert, Heinrich Neubauer, Wolf D. Splettstoesser

**Affiliations:** Hesse State Health Office, Dillenburg, Germany (A.M. Hauri); Public Health Authority Darmstadt-Dieburg, Darmstadt, Germany (I. Hofstetter, J. Eckert); National Reference Laboratory for Tularemia, Munich, Germany (E. Seibold, P. Kaysser, W.D. Splettstoesser); Institute of Bacterial Zoonoses, Jena, Germany (H. Neubauer).; 1 Current affiliation: University Hospital Rostock, Rostock, Germany.

**Keywords:** *Francisella tularensis holarctica*, Germany, airborne transmission, hare hunting, tularemia, zoonoses, research

## Abstract

In November 2005, an outbreak of tularemia occurred among 39 participants in a hare hunt in Hesse, Germany. Previously reported tularemia outbreaks in Germany dated back to the 1950s. We conducted a retrospective cohort study among participants and investigated the environment to identify risk factors for infection. Ten participants had serologic evidence of acute *Francisella tularensis* infection; 1 other participant died before laboratory confirmation was obtained. Presence within 5 meters of the place where disemboweled hares were rinsed with a water hose was the risk factor most strongly associated with infection (risk ratio 22.1; 95% confidence interval 13.2–154.3). Swabs taken at the game chamber and water samples were PCR negative for *F. tularensis*. Eleven of 14 hare parts showed low-level concentrations of *F. tularensis*, compatible with cross-contamination. More than half of case-patients may have acquired infection through inhalation of aerosolized droplets containing *F. tularensis* generated during rinsing of infected hares.

## Abstract 2

Infectious aerosols can contribute to the transmission of tularemia during processing of dead hares.

In the last 50 years, few laboratory-confirmed outbreaks of airborne tularemia have been described. They include outbreaks in workers in sugar cane factories in Ukraine, the Czech Republic, and Austria (*1*–*3*); farmers in Sweden and Finland (*4*,*5*); and residents of the island of Martha’s Vineyard, Massachusetts, USA (*6*). Small clusters and outbreaks with probable common source exposure may have been associated with disturbance of contaminated animal carcasses (*7*–*9*) and dogs with contaminated fur shaking themselves inside houses (*10*,*11*). In Germany, tularemia is rare, with only 184 cases reported during 1955–2004 (*12*). Starting in late 2004, tularemia reemerged, causing repeated outbreaks in nonhuman primates at different animal facilities in central Germany (*13*), followed by rising numbers of human cases in 2005, 2007, and 2008. Here we report a point-source outbreak of tularemia among participants of a hare hunt in Hesse, Germany, in November 2005.

## The Outbreak

On December 1, 2005, Darmstadt health authorities were notified of a laboratory-diagnosed case of tularemia. The patient had participated in a hare (*Lepus europaeus*) hunt on October 29, 2005, and cut 1 finger while disemboweling and skinning hares. On November 2, the patient became ill with fever >40°C, axillary lymphadenopathy, arthralgia, and headache. Initially treated as an outpatient, he was hospitalized November 21 for progressive lymphadenitis and recurrent fever; *Francisella tularensis* infection was diagnosed by lymph node biopsy and specific antibodies. After the Darmstadt-Dieburg Public Health Authority received notification of this index case, that agency initiated an outbreak investigation.

On October 29, 2005, 29 hunters and 10 beaters, who drove hares out of areas of cover, participated in the hunt. Sixty-three hares were shot. Some hares were disemboweled where they were shot; most were later disemboweled and rinsed with a water hose at a hunting lodge. Disemboweled hares were transported to a game chamber and skinned the next day.

## Materials and Methods

### Patients

All participants of the hunt were offered serologic testing. From December 3, 2005, through March 3, 2006, serum was obtained from 29 participants, and DNA was extracted from an affected lymph node of the index case-patient.

Two different case definitions were used. Symptomatic participants of the hunt who fell ill during October 30–November 12, 2005, were defined as confirmed case-patients if they had a single high titer of *F. tularensis*–specific antibodies. We defined a probable case-patient as either an asymptomatic hunt participant with a single high titer of *F. tularensis*–specific antibodies or a hunt participant who had signs and symptoms suggestive of *F. tularensis* infection from October 30 through November 12, 2005, but no laboratory confirmation.

### Retrospective Cohort Study

Starting December 13, 2005, we interviewed hare hunt participants using a standardized questionnaire to determine demographic and clinical details and risk factors for *F. tularensis* infection. For statistical analysis, we combined probable and confirmed cases; all participants who did not fulfill a case definition were included as controls. All analyses were performed with Intercooled STATA 10.0 for Windows statistical software (StataCorp, College Station, TX, USA). Fisher exact test was used to analyze the relationship between categorical variables and the 2-sample Wilcoxon rank-sum test used to analyze the relationship between numeric data and the categorical outcome.

### Environmental Investigation

**Figure 1:**
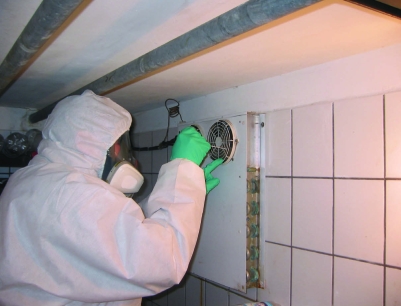
Sampling at the game chamber, Hesse, Germany, December 2005.

Starting in early December 2005, we visited the outbreak area 3 times. We obtained data on elevation, regional mean annual air temperature, precipitation, and sunshine hours (1961–2004) from the Federal Meteorological Service (Offenbach am Main, Germany). Water samples were obtained from a small creek near the hunting lodge and from the water hose used to rinse disemboweled hares. Additionally, 28 samples were taken at the game chamber (Table 1; Figure 1). All samples were stored at 4°C.

**Table 1:** Type of environmental samples taken and results of testing for *Francisella* *tularensis*, Germany, 2005*

Type	No. samples	Origin	Results		
			PCR	Culture	Ag detection (LPS)
Water	2	Creek, water hose	Neg	Neg	Neg
Swabs	16	Game chamber	Neg	Neg (4/4)	–
Hare fur, insects	3	Game chamber	Neg	–	–
Liquid samples (flush)	9	Game chamber	Neg	–	-
Frozen parts of hares received from 9 different households (muscle, bone marrow, fluids recovered during thawing)	14	12–14 hares (*Lepus europaeus*), shot 2005 Oct 29	Pos 11*/14	Neg (6/6)	Pos. 6†/14
Liver/spleen samples	29	15 hares, 1 nutria (*Myocastor coypus*), shot 2005 Dec 12	Neg	–	–
Organs of hares (liver, spleen, whole blood)	72	24 hares, shot 2006 Jan 7 and Jan 14	Neg‡	–	–

*For all parts, tissue, bone marrow, and fluid from thawing were tested. Samples were considered positive when >2 materials were repeatedly positive by 2 different PCRs. Ag, antigen; LPS, lipopolysaccharide; Neg, negative; Pos, positive. †Samples also positive by PCR. ‡PCR inhibition noticed for 19/24 blood samples.

Deep frozen parts from 12–14 hares shot during the initial hunt on October 29 were recovered from different households. Additional animals were shot in the same hunting area on December 6, 2005, and January 7 and 14, 2006. In January 2006, all frozen samples were transported on dry ice to a microbiologic laboratory.

### Laboratory Methods

#### Direct Detection of *F. tularensis*

All animal samples were stored at −20° C until preparation for PCR, antigen detection, or culture. Specimens of spleens, livers, bone marrow, and muscle tissue of the animals were homogenized as described recently (*13*) and tested for *F. tularensis*–specific lipopolysaccharide (LPS) using a capture ELISA (*14*) or an immunochromatographic column assay (ABICAP, Senova, Jena, Germany). Purified DNA was prepared from tissue homogenates, blood, and water samples and from fluids obtained during thawing of the hare samples by using the QIAamp Tissue kit (QIAGEN, Hilden, Germany).

PCR amplification and product detection were performed in a LightCycler instrument (Roche, Mannheim, Germany) by using a commercially available real-time PCR kit (TibMolBiol, Berlin, Germany) for the detection of a specific nucleotide sequence within the 16S rRNA gene of *F. tularensis* (*15*). Additionally, real-time PCR protocols targeting the *tul4* (*16*), *iglC*, *ISFtu2*, or *fopA* gene were performed (*17*). Each run included positive and negative controls. For subspecies identification, a conventional PCR protocol employing primers flanking the RD1 region of *F. tularensis* was used (*18*). To prove the presence of *F. tularensis*–specific DNA in hares showing a low signal in the screening PCR, we performed amplification of a 16S rRNA gene target followed by sequencing of the fragment.

#### Serum Samples and Culture Recovery of *F. tularensis*

Water samples; swab samples; and spleen, liver, and bone marrow homogenates were cultured on cysteine heart agar supplemented with 9% sheep blood, Columbia blood agar, McConkey agar, and modified Thayer-Martin medium containing antimicrobial drugs (Merck, Darmstadt, Germany). Culture plates were incubated at 37°C for 10 days and investigated daily for bacterial growth (*13*). Serum from 29 participants was examined for *F. tularensis*–specific anti-LPS antibodies by a qualitative screening ELISA and confirmed by immunoblot (*18*).

## Results

### Patients: Clinical Characteristics and Laboratory Results

**Figure 2:**
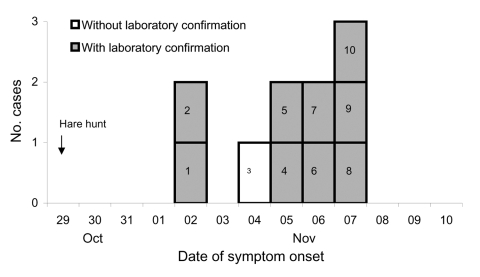
Tularemia cases (n = 10), by symptom onset, County of Darmstadt-Dieburg, Germany, October–November 2005.

Characteristics of 9 hare hunt participants met the definition of a confirmed case; 2 participants had characteristics that met the definition of a probable case. The median age of case-patients was 55 years (range 11–73 years); all were male. Illness onsets ranged from November 2 through November 7 (Figure 2). One probable case-patient (no. 3 in Figure 2) fell ill with high fever, myalgia, and clinically diagnosed bilateral pneumonia; he had chronic heart failure and died during the second week of illness despite treatment with moxifloxacin. Neither specific antibodies nor *F. tularensis*–specific DNA could be detected in a serum specimen taken 8 days after illness onset. The second probable case-patient was asymptomatic but had high levels of anti-LPS–specific antibodies (immunoglobulin [Ig] M 32,000; IgA 32,000; IgG 8,000), suggesting a recent subclinical infection. Antibody titers of the 9 confirmed case-patients ranged from 64,000 to >256,000 (negative <500). All 9 showed a specific IgG, IgA, and IgM immune response, all were medically attended, and 1 was hospitalized. They reported fever >38.5°C (range 38.5°C–40.6°C) (8 persons), chills (6), headache (5), weight loss (5), myalgia (5), enlarged lymph nodes (3), and coughing (1). None reported sore throat or pneumonia. Two case-patients had an ulceroglandular form of tularemia: the index patient (case-patient 1 in Figure 2) had cut his finger while skinning hares; the other (case-patient 6) had scratched his finger before the hunt.

PCR of an affected lymph node specimen and sequencing of the amplificate indicated *Francisella* infection. Real-time PCR (targets 16S rRNA gene, *tul4* gene) confirmed the presence of *F. tularensis*–specific DNA within the sample. Partial amplification of the RD 1 region identified a 923-bp fragment considered to be specific for subspecies *holarctica* (*18*). Several attempts to amplify *F. tularensis* DNA fragments from serum of case-patient 3 were unsuccessful.

### Retrospective Cohort Study

The analysis included data for 10 of the 11 case-patients and all 28 controls (Table 2). Presence within 5 meters of where disemboweled hares were rinsed was the risk factor most strongly associated with infection. Case-patient 3, who died, was not included in the cohort study; however, he was reported to have disemboweled hares within 5 meters of the area where disemboweled hares were rinsed. Hares were disemboweled and rinsed at the hunting lodge during the lunch break and in the afternoon after the hunt. Ten case-patients were at the lodge at the end of the hunt; 7 were at the lodge during the lunch break. In case-patient 6, who was not at the hunting lodge in the afternoon, ulceroglandular tularemia developed. The asymptomatic hunter (no. 11) had disemboweled ≈12 hares at a distance 8–10 meters from the place where hares were rinsed. One person present at the hunting lodge, whose laboratory tests were negative for *F. tularensis*, reported that although he had been within 5 meters of the area where disemboweled hares were rinsed, he preferred to keep some distance from the group that were handling the hares.

**Table 2:** Attack rates among exposed and nonexposed hare hunters, according to potential risk factors for *Francisella tularensis* infection, Germany, 2005*

Potential risk factor	Exposed				Not exposed			RR (95% CI)	p value
	No. cases	No. hunters	Attack rate, %		No. cases	No. hunters	Attack rate, %		
Hunted	8	27	29.6		2	10	20.0	1.5 (0.4–5.8)	0.45
Had direct contact with dead hares	10	34	29.4		0	4	0	–	0.2
Wore gloves during contact with dead hares	2	5	40.0		8	21	38.1	1.5 (0.4–4.9)	0.6
Injured skin	4	8	50.0		5	25	20.0	2.5 (0.9–7.1)	0.12
Disemboweled hares	7	11	63.6		3	27	11.1	5.7 (1.8–18.2)	0.002
Injured while disemboweling hares	1	2	50.0		9	36	25.0	2.0 (0.5–8.9)	0.46
Rinsed hares at the lodge	4	6	66.7		6	32	18.8	3.6 (1.4–8.9)	0.03
Presence within 5 m of where disemboweled hares were rinsed	9	11	81.8		1	27	3.7	22.1 (3.2–154.3)	<0.0001
Skinned hares	5	9	55.6		5	29	17.2	3.2 (1.2–0.7)	0.04
Injured while skinning hares	1	1	100		9	37	24.3	4.1 (2.3–7.3)	0.26
Had contact with raised dust	4	5	80.8		6	33	18.2	4.4 (1.9–10.3)	0.01
Had contact with puddle or ditch water	0	1	0		10	37	27.0	–	0.74
Received tick bite on the hunting day	0	0	–		10	38	26.3	–	–
Consumed hares hunted on October 29, 2005	0	4	0.0		10	34	29.4	–	0.28
Attended the common lunch/supper	9	35	25.7		1	3	33.3	0.8 (0.0–4.2)	0.61
Stayed abroad in October 2005	4	7	57.1		6	30	20.0	2.9 (1.1–7.5)	0.07

*RR, risk ratio; CI, confidence interval. Totals vary because of answers of “do not know.”

### Environmental Investigations

The outbreak area has several ecologic characteristics that were shown to correlate with high numbers of tularemia foci in the Czech Republic (Table 3). According to the hunters, all hares shot during the hunt on October 29 appeared healthy and showed no macroscopic signs of systemic infection (e.g., enlarged liver or spleen). No die-off of hares or rodents was observed in the region.

**Table 3:** Ecologic characteristics of the outbreak area in Germany and of natural habitats correlated with a high number of tularemia foci in the Czech Republic

Characteristic	Outbreak area, Germany, 2005	Natural habitats in the Czech Republic with high numbers of tularemia foci (*19*)
Elevation above sea level	88–112 m	Up to 200 m
Mean annual air temperature	10.0°C (1994–2004)*	8.1–10.00°C
Mean annual precipitation	673.5 mm (1994–2004)*	450–700 mm
Habitat	Single trees along a creek, alluvial forest-like field biotope, surrounded by areas of intensive agriculture	Alluvial forests, field biotopes
Mean annual sunshine duration	1,685 h (1994–2004)*	2,001–2,200 h

*Ten-year period preceding the year in which the outbreak occurred.

Samples taken in the game chamber and of the water were negative for *F. tularensis*, whereas samples taken from 11 of 14 parts of hares from the initial hunt were positive (Table 1). Six of these samples were additionally positive for *F. tularensis*–specific LPS.

## Discussion

We investigated an outbreak of tularemia after a hare hunt in Hesse, Germany, for which epidemiologic, clinical, and microbiologic data indicate an airborne origin. The results of the cohort study support this hypothesis on the basis of the association between case status and presence within 5 meters of the location where disemboweled hares were rinsed. During the afternoon session of disemboweling and rinsing hares, 10 of the 11 case-patients were at the hunting lodge; aerosolization of infectious particles may have been limited to this session. Three case-patients, among them the patient who did not participate in the afternoon session, had a glandular or ulceroglandular form of tularemia. They may have acquired infections through skin lesions. The absence of cutaneous lesions or lymphadenopathy in the remaining 8 patients makes a cutaneous route of infection less likely than a respiratory route. The low incidence of respiratory symptoms among our patients is in agreement with findings from previous airborne outbreaks that involved patients infected with the less virulent subspecies *F. tularensis* *holarctica*, in which only a minority of case-patients had symptoms suggestive of pneumonia (*8*,*9*).

Two hunters met the probable case-patient definition. The asymptomatic hunter (no. 11) disemboweled hares at a distance from the group. Severity of clinical tularemia has been correlated with infectious dose (*20*), and this hunter might have been exposed to a smaller pathogen load or exposed on another recent occasion. Case-patient 3 died during the second week of illness. Antibodies against *F. tularensis* in most patients appear 6–10 days after onset of symptoms (*21**,**22*). Serum available for testing from case-patient 3 was from his eighth day of illness; hence, it was possibly taken before a measurable antibody response developed. We further cannot exclude the possibility that the 10 asymptomatic participants who did not undergo laboratory testing had to be considered as probable case-patients if they provided a serum sample.

Detection of *F. tularensis* in hare specimens, including bone marrow specimens, and lack of *F. tularensis* detection in samples of the water system used to rinse hares suggest infection of the hares. One or more infected hares, still bloody and wet, may have cross-contaminated additional hares during further processing, e.g., during transport to and storage at the game chamber. All samples taken in the game chamber showed negative results. Case-patient 3 had cleaned the game chamber thoroughly with a pressure washer, possibly exposing himself to a high pathogen load.

Small clusters and outbreaks of airborne tularemia have been associated with hares or rabbits (*7*–*11*). However, most cases of tularemia associated with hares are of the ulceroglandular or glandular form (*1*,*22*). In a protracted outbreak in Spain, 97% of patients reported previous contact with hares; 71% of these had a glandular or ulceroglandular form of disease (*23*). Of 577 case-patients treated at a clinic in Czechoslovakia, 194 had direct contact with hares, and an (ulcero) glandular form of disease developed (*1*). Different frequencies of the diverse clinical forms of tularemia suggest differences in the main route of pathogen acquisition.

In the retrospective cohort study, presence within 5 meters of the place where disemboweled hares were rinsed was the risk factor most strongly associated with infection. Washing of contaminated produce was found to be an effective mechanism of generating infectious aerosols in tularemia outbreaks in sugar beet factories (*1*–*3*), and rinsing >1 hares infected with *F. tularensis* was the most probable way by which an infectious aerosol was generated. However, we cannot exclude the idea that an infectious aerosol was formed through further hare manipulating activities, e.g., transport.

Previous outbreaks in Germany date back to the 1950s, with the last case reported in the outbreak area in 1957 (*24*). Environmental characteristics of natural foci of tularemia persisting over >30 years have been described (*19*,*25*). The outbreak region in Germany shares several features favoring the occurrence or persistence of *F. tularensis* in the environment. Recently, the presence of *F. tularensis* in trapped rodents (2.1%) and in water samples from this hunting area was directly confirmed, and >10% of rodents in several German regions not previously considered as endemic foci were infected (*19*). In addition, *F. tularensis* was repeatedly detected in 22 hares from 5 federal states (Bavaria, Hesse, Baden-Wuerttemberg, Thuringia, and Lower Saxony) after improved diagnostic tools (real-time PCR) had been applied complementary to standard 48-h bacterial cultivation (W.D. Splettstoesser et al., unpub. data). Together with results obtained from serologic studies in the German population (*26*), the outbreak reported here suggests that tularemia has either reemerged in Germany or is seriously underreported.
